# Determinants of Patients’ Perception of Primary Healthcare Quality: Empirical Analysis in the Brazilian Health System

**DOI:** 10.3390/healthcare13080857

**Published:** 2025-04-09

**Authors:** Maria Luisa de Oliveira Collino Antiga, Bruna Leão Freitas, Roxanne Brizan-St. Martin, Althea La Foucade, Flavia Mori Sarti

**Affiliations:** 1 School of Arts, Sciences and Humanities, University of São Paulo, São Paulo 03828-000, Brazil; 2 Faculty of Social Sciences, The University of the West Indies, St. Augustine, Trinidad and Tobago; roxanne.stmartin@oecs.int (R.B.-S.M.); althea.lafoucade@sta.uwi.edu (A.L.F.)

**Keywords:** healthcare management, health system, healthcare quality, healthcare effectiveness, patient satisfaction

## Abstract

**Background/Objectives**: Primary healthcare (PHC) plays a central role in the promotion of universal healthcare coverage within the Brazilian health system. Nevertheless, inequalities across municipalities represent substantial barriers to achieving equity in access to health, particularly due to disparities in the quality of healthcare delivered to patients. Thus, the study aimed to investigate factors associated with perception of PHC quality among adult individuals using private and public facilities within the Brazilian health system. **Methods**: The empirical approach was based on quantitative analysis of cross-sectional data from five nationally representative surveys conducted by the Brazilian Institute for Geography and Statistics (Instituto Brasileiro de Geografia e Estatística, IBGE) in 1998, 2003, 2008, 2013, and 2019. Pairwise comparisons and marginal analyses allowed for the assessment of differences in patients’ perception of healthcare quality according to source of funding and type of healthcare quality. A logistic regression model was estimated to identify factors associated with the perception of good quality of care. Model discrimination, calibration, and goodness-of-fit were assessed to ensure the robustness of analyses. **Results**: The results indicate that patients’ satisfaction was positively associated with level of implementation of the national program based on payment for performance in public healthcare facilities, PMAQ-AB (OR = 3.376; *p* < 0.001), self-assessment of good health status (OR = 3.209; *p* < 0.001), and healthcare financed through health insurance (OR = 2.344; *p* < 0.001). Contrarily, receiving healthcare in a public facility (OR = 0.358; *p* < 0.001) was negatively associated with the evaluation of good quality. **Conclusions**: The findings showed that patients’ perception of quality of care presents significant associations with patients’ health characteristics, healthcare funding source, and implementation of the PMAQ-AB. Furthermore, patients generally perceived lower healthcare quality in public facilities. The study indicates the need for evidence-based decision-making in public policies of health, particularly regarding further advances in payment for performance programs designed to foster improvements in quality of care within public PHC facilities in Brazil.

## 1. Introduction

Primary healthcare (PHC) encompasses strategies for health promotion and disease prevention based on the coordination of flows of information, assistance, and patients within national health systems worldwide [1,2]. The lack of timely access to high-quality PHC compromises short- and long-term health outcomes at the population level, increasing the utilization of emergency care, generating dissatisfaction among patients, and expanding costs in the health system [3,4,5,6].

The Brazilian Unified Health System (Sistema Único de Saúde, SUS) was established to promote universal coverage based on the provision of comprehensive healthcare without charge to patients. The SUS is publicly funded through taxes [7], being predominantly focused on the expansion of PHC coverage [8,9,10]. Furthermore, the promotion of healthcare quality at the primary level was emphasized in 2011 through the National Program for Improving Primary Care Access and Quality (Programa Nacional de Melhoria do Acesso e da Qualidade da Atenção Básica, PMAQ-AB) [11].

The PMAQ-AB was based on the distribution of financial incentives to municipalities willing to participate in the program, according to scores obtained in the scheme of payment for performance. The implementation of the PMAQ-AB occurred in three consecutive cycles between 2011 and 2019, addressing diverse performance measures related to infrastructure and coverage, in addition to self-assessment of PHC teams and evaluation from external stakeholders [11,12,13,14,15].

The PMAQ-AB was recently replaced by the Previne Brasil program (PB) at the end of the third cycle in 2019. The PB is based on changes in the criteria for the distribution of financial incentives: weighted capitation of PHC coverage, payment for performance, and incentives for strategic programs. However, its implementation has been compromised due to consecutive postponements in the distribution of financial incentives, resulting in municipalities’ withdrawal from the program [16].

Nevertheless, inequalities in infrastructure across Brazilian municipalities still represent substantial barriers to universal healthcare coverage, particularly due to disparities in quality and the effectiveness of PHC provided to the population [2,3,8,17]. Yet, there is gap in the literature on the associations between patients’ perceptions of the quality and effectiveness of PHC at the national level in Brazil. Previous studies have focused on small-scale samples of patients [18,19,20,21] or have relied on analyses of specific indicators of the PMAQ-AB at a local level [22,23,24].

The scarcity of population-representative surveys including patient-centered data on healthcare experiences hinders further advances in the consolidation of evidence on the connections between patients’ satisfaction with PHC, effectiveness, and other determinants of access and utilization of healthcare in developing countries, especially in Latin America [25,26,27]. Studies focusing on healthcare quality through patients’ views generally rely on qualitative analysis at a local level [28] or data from surveys with limited sample size in developed or developing countries, particularly in the Middle East or Asia [29,30,31]. An evidence-gap map on health systems’ performance in PHC among low- and middle-income countries showed lack of studies focusing on Latin American countries [29].

National health systems pursuing universal healthcare coverage strive to promote equality in health through a timely supply of accessible, affordable, and effective healthcare [32]. Health inequalities linked to social determinants range from lack of access to basic sanitary infrastructure and healthcare facilities to disparities in healthcare quality and effectiveness. Inequalities generally concentrate among individuals with higher exposure to health risks and higher socioeconomic vulnerability, i.e., inverse care law [33]. Furthermore, the absence of high-quality healthcare tends to generate vicious cycles of disease, unemployment, and intergenerational transmission of poverty [34].

The theoretical debate on healthcare quality encompasses extensive literature on the definitions, measurement, and indicators to address its multidimensional attributes at the individual and social levels. The classical Donabedian model still represents one of the main approaches for the evaluation of quality in health systems, examining the validity of indicators for assessment of quality in three domains of supply: structure, process, and outcomes [35,36,37]. Considering the complexity of the measurement of quality due to its nature of value judgments on healthcare outputs [38,39,40,41,42,43,44,45,46,47], diverse definitions have been proposed to capture the capacity to achieve desirable health outcomes at individual or population levels [38].

Yet, a major part of the literature concurs that the assessment of quality should rely on judgments of health system stakeholders [35]. Donabedian’s model [35,36] emphasizes patient-centered health systems, highlighting the value of patients’ perception for the measurement of healthcare quality [35]. Thus, the concept of quality adopted in the present study corresponds to acceptability, i.e., the extended notion of patients’ satisfaction based on subjective evaluation at the individual level in response to the healthcare experience [26,48,49,50].

Furthermore, the study proposes to explore associations between patients’ satisfaction in relation to individual, household, health service, and policy-level characteristics. At the policy level, national health systems focusing patient-centered strategies usually rely on the implementation of programs for improvement of quality, including incentives on healthcare performance [38]. Therefore, the framework adopted in the study includes information on adherence to the PMAQ-AB, considering its focus on payment for performance at the primary care level.

Regarding health service characteristics, the study explores indicators of the structure and process of healthcare supply: type of healthcare facility, source of financing, and healthcare effectiveness. The concept of healthcare effectiveness adopted in the study refers to the capability of generating desirable health outcomes within a reasonable timeframe [36,40], i.e., the ability to address patients’ healthcare demands through the resolution of their health issues [41,51,52,53,54].

**Figure 1:**
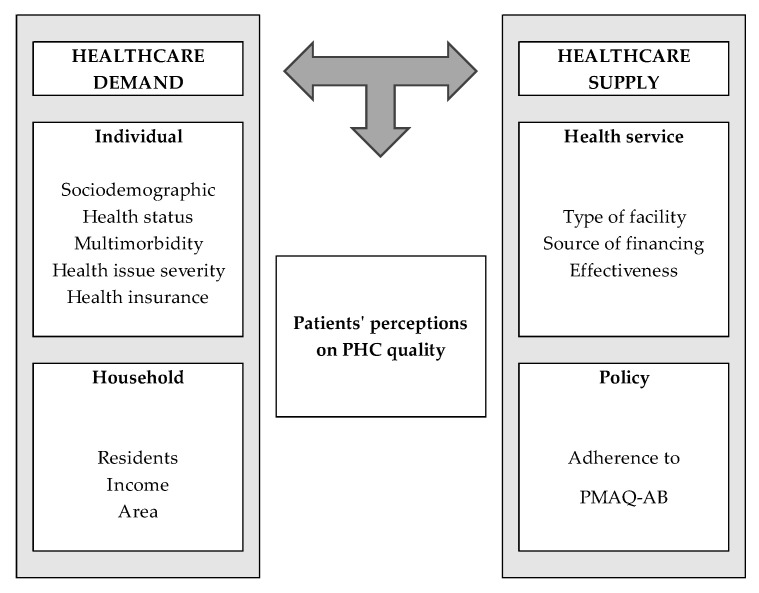
Analytical framework of the study.

Finally, individual and household characteristics included in the analytical framework of the study allows for the identification of the contextual and structural determinants of healthcare demand that may interfere in the perception of healthcare quality [55,56]. The analytical framework proposed to guide the empirical strategy for the identification of determinants of patients’ satisfaction with PHC in Brazil relies on the interrelations among elements of healthcare demand and supply, as represented in Figure 1.

The study addresses the gap in the literature referring to the empirical analysis of individual, household, health service, and policy-level determinants of patients’ perception of PHC quality in Brazil, through examination of data representative at the national level in the largest developing country in Latin America [27,28,29,30,31].

## 2. Materials and Methods

### 2.1. Study Design

The study comprises quantitative analysis of individual-level data from five cross-sectional surveys representative at the population level conducted by the Brazilian Institute for Geography and Statistics (Instituto Brasileiro de Geografia e Estatística, IBGE) in 1998, 2003, 2008, 2013, and 2019.

### 2.2. Datasets

The study aimed to investigate factors associated with the perception of PHC quality among adult individuals using private and public facilities within the Brazilian health system, using nationally representative surveys data collected between 1998 and 2019.

The datasets of the study were three National Household Sample Surveys (Pesquisa Nacional por Amostra de Domicílios, PNAD) referring to 1998, 2003, and 2008, and two National Health Surveys (Pesquisa Nacional de Saúde, PNS) referring to 2013 and 2019. PNAD databases comprise annual surveys conducted by IBGE, based on a probabilistic sampling process in three stages (municipalities, census tracts, and households), including supplementary data on health status and health system utilization in 1998, 2003, and 2008. PNS databases refer to regular surveys conducted by IBGE on health status and health system utilization in 2013 and 2019, based on a probabilistic sampling process in three stages (census tracts, households, and individuals).

The complex sample design adopted in the PNAD and PNS surveys allows expansion of the sample for representation of the population according to geographical areas (national, regional, and state levels). Municipalities and census tracts included in the sample selection were drawn based on probability proportional to their population. The national-based registry of the Brazilian population was used to invite individuals in households selected to participate in the PNAD and PNS surveys. Uninhabited/unoccupied/abandoned households were excluded from the samples of the PNAD and PNS surveys. Inhabited households and their respective household members drawn in the PNAD sample selection, independently of age or gender, were eligible to participate in the surveys, whereas sample selection in the PNS surveys included only household members aged ≥18 years.

Trained interviewers administered the questionnaire, which mainly featured closed-ended questions. Information obtained from individuals was organized into anonymized datasets, which are publicly available on the platform of the IBGE corresponding to PNAD (https://www.ibge.gov.br/estatisticas/sociais/populacao/19897-sintese-de-indicadores-pnad2.html?=&t=microdados accessed on 5 April 2025) and PNS (https://www.ibge.gov.br/estatisticas/downloads-estatisticas.html accessed on 5 April 2025). The protocols of the surveys were approved by the Brazilian National Research Ethics Commission, following the ethical principles of the Helsinki Declaration.

The present study was based on the selection of individual-level data compatible across surveys, using information reported by adult individuals (≥18 years old) to ensure comparability of samples surveyed in PNAD and PNS. The selection of variables compatible across the PNAD and PNS surveys was based on similarity in wording of questions and similarity of categories presented in multiple choice questions. Thus, variables regarding domains of fertility, migration, job and employer characteristics, and healthcare expenditures were excluded from the PNAD surveys.

Variables excluded from the PNS surveys referred to detailed description of health status and healthcare in the following domains: hearing and visual impairments, mental deficiencies, quality of sleep, oral health, use of alternative medicine practices, markers of healthy and unhealthy food consumption patterns, physical activity, tobacco use, pediatric and maternal care, anticonception practices, and chronic diseases management.

The information was organized into a single database to allow analysis of associations between patients’ view on healthcare quality and effectiveness of care according to the type of healthcare facility. Additional information regarding the implementation of the PMAQ-AB at the municipal and national level was obtained from the Brazilian Ministry of Health, being incorporated into the dataset to examine the potential influence of the implementation of the program on the perception of healthcare quality at the individual level.

### 2.3. Variables

The variables selected from the PNAD and PNS datasets were directly comparable across surveys. The target variable of the study refers to patients’ perception of the quality of healthcare provided at the primary level (binary: regular or less and good or very good). The variables of interest included individual, household, health service, and policy-level characteristics, in addition to control variables (Table 1):

- Individual characteristics: sex (binary: male, female), age (continuous: years), skin color/ethnicity (categorical: black, brown, indigenous, white, and yellow), educational attainment (continuous: years of education), occupational status (binary: unemployed and employed), health status (binary: regular or less and good or very good), diagnosis of multimorbidity (binary: no and yes), health issue severity (binary: no and yes), health insurance ownership (binary: no and yes);
- Household characteristics: household residents (discrete: individuals living in the household), household income per capita in adult equivalents (continuous: dollars in 2022 purchase power parity, PPP), area of residence (binary: rural, and urban);
- Health service characteristics: type of healthcare facility (binary: public, and private), source of healthcare financing (categorical: health insurance, out of pocket, and SUS), healthcare effectiveness (proportion of healthcare visits required to solve health issues during the last two weeks);
- Policy-level characteristics: adherence to the PMAQ-AB (binary: low and high adherence to the payment for performance program);
- Control variables: state (categorical: 26 states and the federal capital), year of the survey.

The following variables were based on self-declaration of individuals into predefined categories: skin color/ethnicity, health status, health issue, and characteristics of primary healthcare utilization during the last two weeks. Skin color/ethnicity was based on traditional categorization adopted in Brazilian surveys using five categories—black, brown, indigenous, white, and yellow, being converted into five binary variables. Health status was based on self-assessment of health conditions based on 5-point Likert scale (very poor, poor, regular, good, and very good), converted into binary variables referring to very poor, poor, and regular health status (0) and good and very good health status (1).

Primary healthcare utilization was based on the need to access health services within the two weeks previous to the survey, excluding demand for hospital and emergency care. Individuals accessing PHC facilities were questioned on the characteristics of services, including perception of quality, healthcare visits required to solve the health issue, source of funding, and type of healthcare facility.

The health issue severity was based on healthcare provided in the PHC facility, converted into binary variables referring to visits to physicians or other health professionals, medication, vaccination, and other preventive care (0) and ambulatory surgery or referral to hospitalization (1). Perception of PHC quality was based on the assessment of the acceptability of services through a 5-point Likert scale (very poor, poor, regular, good, and very good), converted into binary variable referring to perception of very poor, poor, and regular healthcare quality (0) and good and very good healthcare quality (1).

Healthcare effectiveness was based on the proportion of healthcare visits required until the resolution of the health issue considering the period within the two weeks before the survey; i.e., fewer PHC visits needed to solve health demands of patients corresponded to higher effectiveness. The source of funding for health services was declared by individuals according to three categories: health insurance, out-of-pocket disbursement, or government expenditures within the SUS. The type of healthcare facility was declared by individuals according to two categories: public or private.

Household income per capita in adult equivalents was based on the aggregation of income from household residents divided by adult equivalents in the household, estimated through the adult equivalent scale (*e_j_*) using 0.75 weight for individuals ≤14 years old (Equation (1)):

(1) $$e_{j}={\left(A_{j}+{\Phi \cdot K}_{j}\right)}^{\theta }$$

where *A_j_* = adults in the household *j*; *K_j_* = children ≤14 years old in the household *j*; and *Φ* = *θ* = 0.75, weight defined in the literature [57]. In addition, monetary values were updated to the period of reference of December 2022, being converted into dollars in purchase power parity (PPP) to allow comparisons at the international level, using the corresponding PPP conversion factor available at the platform of the World Bank [58].

The variable on the adherence to PMAQ-AB was based on reports referring to transferences of federal funds for the municipalities according to their level of adherence to the program, considering the intersection between the period of implementation of the program (2011–2019) and years of PNS surveys (2013 and 2019).

Data on the proportion of municipalities with adherence to the program, obtained on the platform of the Brazilian Ministry of Health (https://www.gov.br/saude/pt-br/composicao/saps/pmaq accessed on 5 April 2025), was incorporated into the individual-level dataset to comprise indicators of potential incentives to promote PHC quality at the state level, considering payments for performance implemented in healthcare facilities fulfilling the requirements of the PMAQ-AB.

### 2.4. Statistical Analyses

Descriptive statistics were estimated using the mean and standard error for continuous variables and frequencies for categorical variables. Pairwise comparisons and marginal analyses were conducted to assess differences in patients’ perception of PHC quality across sources of healthcare funding and types of healthcare facilities, comparing the private and public sectors, considering the prominent role of government investments in the promotion of healthcare quality in Brazil. Additional pairwise comparisons between independent variables in relation to the dependent variable were included in the Appendix A.

The estimation of a logistic regression model was conducted to investigate factors associated with patients’ satisfaction with PHC (outcome variable), particularly focusing on healthcare effectiveness (variable of interest), sociodemographic and health characteristics of individuals, and household characteristics (Equation (2)):

(2) $$\log\left(\frac{\pi_{ijt}}{1-\pi_{ijt}}\right)=\beta_{0}+\beta_{1}\cdot S_{ijt}+\beta_{2}\cdot H_{ijt}+\beta_{3}\cdot {HH}_{jt}+\beta_{4}\cdot C_{jt}$$

where *π_ijt_* = probability of perception of good healthcare quality for individual *i* in household *j* in the period *t*; *S_ijt_* = matrix of sociodemographic characteristics of individual *i* in household *j* in the period *t*; *H_ijt_* = matrix of health characteristics of individual *i* in household *j* in the period *t*; *HH_jt_* = matrix of characteristics of household *j* in the period *t*; and *C_jt_* = matrix of control variables referring to the state of residence, year of the survey, and interaction between state of residence and year of the survey.

The statistical analyses were performed using the software Stata, version 17.0, adopting a significance level of *p* < 0.05. Manual stepwise selection of variables was adopted to identify relevant subsets of predictors for the logistic regression model. Post-estimation tests on discrimination, calibration, and goodness-of-fit were estimated for diverse specifications of the model to ensure robustness of the analyses, using the area under the receiver operating characteristic (ROC) curve, Hosmer–Lemeshow statistic, and Pearson goodness-of-fit test, respectively.

## 3. Results

The major part of the individuals surveyed were female, approximately 47 years old, self-declaring as having white or brown skin color, and living in urban areas in the southeast region. Educational attainment increased from approximately 7 to 10 years of schooling, and household income per capita raised from 933.89 to 1277.08 dollars PPP in the period from 1998 to 2019 (Table 2).

A demographic transition occurred throughout the period in the country, being shown through the increase in mean population age, whereas household size reduced from approximately four to three residents in the period from 1998 to 2019. The proportion of employed persons increased in the period between 1998 and 2008; however, a decrease occurred in subsequent years (Table 2).

There was an increase in the proportion of individuals indicating good health status, individuals diagnosed with multimorbidity between 1998 and 2019. In addition, the proportion of individuals with health insurance decreased slightly from 1998 to 2013 (Table 3).

The major part of the population received healthcare in public facilities from 2003 onwards, being financed through government expenditures within the SUS throughout the period from 1998 to 2019. The general perception of PHC quality was high among individuals in the Brazilian population, and individuals identified high healthcare effectiveness at primary level (Table 3).

Differences in perception of quality between funding source and type of healthcare facility showed significantly higher margins in favor of the private sector, presenting lower contrast in patients’ views in 2013 and higher values in 2019 (Table 4). Results of pairwise comparisons between the dependent variable and remaining independent variables are shown in Appendix A (Table A1).

**Figure 2:**
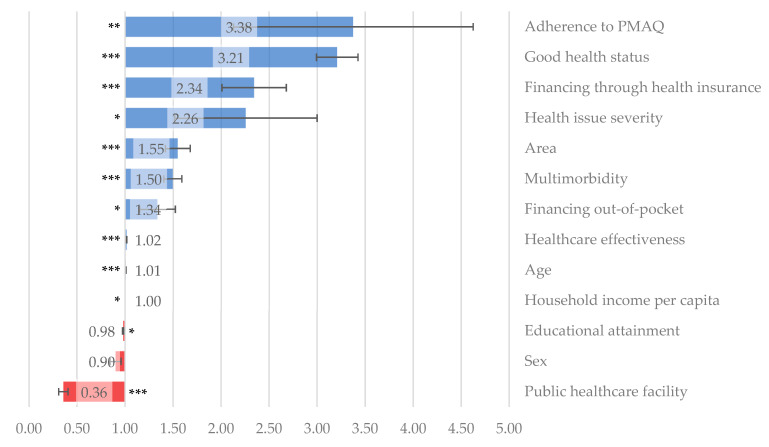
Odds ratios and standard errors for predictors of primary healthcare quality. Brazil, 1998–2019. Obs.: *** *p* < 0.001; ** *p* < 0.01; * *p* < 0.05.

Odds ratios of the logistic regression model showed a higher positive association of healthcare quality in relation to adherence to the PMAQ-AB (OR = 3.376, 95% CI 1.635–6.971, *p* < 0.01), patients’ good health status (OR = 3.209, 95% CI 2.813–3.661, *p* < 0.001), and healthcare financing through health insurance (OR = 2.344, 95% CI 1.772–3.102, *p* < 0.001) (Table 5 and Figure 2). Furthermore, there was a higher probability of satisfaction with PHC among patients diagnosed with multimorbidity (OR = 1.497, 95% CI 1.323–1.694, *p* < 0.001), health issue severity (OR = 2.258, 95% CI 1.185–4.301, *p* < 0.05), financing healthcare through out-of-pocket disbursements (OR = 1.337, 95% CI 1.018–1.757, *p* < 0.05), and higher healthcare effectiveness (OR = 1.017, 95% CI 1.013–1.021, *p* < 0.001) (Table 5 and Figure 2). Contrarily, using public healthcare facilities (OR = 0.358, 95% CI 0.275–0.467, *p* < 0.001) presented a significant probability of a lower perception of healthcare quality (Table 5 and Figure 2).

## 4. Discussion

The present study showed distinctive contributions to the literature on determinants of patients’ satisfaction with PHC by proposing the empirical analysis of individual, household, health service, and policy-level characteristics among adult individuals using private and public facilities within the Brazilian health system from 1998 to 2019. The findings show that the major part of individuals identified good PHC quality, contrasting with previous studies showing a perception of low quality of services among Brazilian patients, particularly due to a lack of infrastructure [3,13,59,60]. Nevertheless, other studies focusing on the quality of PHC provided by multiprofessional teams through regular household visits within the Family Health Strategy program (Estratégia de Saúde da Família, ESF) indicated higher patient satisfaction in comparison to traditional primary healthcare models in Brazil [18,20].

Considering that the major part of the population in PNAD and PNS surveys accessed PHC through the SUS, the evaluation of quality may be linked to preventive care provided within the Family Health Strategy, thus positively influencing trends in patients’ perception of PHC quality provided at public healthcare facilities. Yet, patients’ views on PHC quality were marginally higher among individuals accessing private facilities in comparison to individuals accessing public facilities, consistent with previous findings in the literature [61,62,63]. Evidence from other countries shows contradictory findings: one study in Hong Kong showed higher satisfaction with healthcare in private facilities [64], whereas one study in Malta indicated a lack of difference between patients attending public or private facilities [65].

The quality of care was positively associated with high adherence to the PMAQ-AB, the national program based on payment for performance for Brazilian municipalities, implemented between 2011 and 2019. PMAQ-AB incentives were linked to the assessment of diverse PHC indicators, including certification of multiprofessional teams within the Family Health Strategy program [16,66]. Previous studies indicated that PMAQ-AB resources were distributed to healthcare workers and investments in infrastructure to ensure suitable labor conditions [67], thus comprising potential motivation for the promotion of quality and effectiveness among PHC professionals.

However, incremental changes in PHC policy design were implemented throughout the last decade, including the extinction of the PMAQ-AB in 2019, followed by the failure of the implementation of the successor Previne Brasil program [16,66]. The failure in maintaining programs based on payment for performance within the Brazilian health system may compromise the advances in primary healthcare access, coverage, and health outcomes registered during the first decades of the 21st century [68]. Therefore, the findings of this study comprise valuable evidence in favor of reinstatement of the PMAQ-AB or improvements in the implementation of payment for performance within the current Previne Brasil, considering the substantial effects on patients’ satisfaction. Further advances in design and implementation of financial incentives linked to PHC outcomes should support improvements in the perception of PHC quality among individuals receiving healthcare in public facilities.

Other factors positively associated with healthcare quality in the present study were self-perception of good health status, healthcare effectiveness, and private sources of healthcare funding (out of pocket or health insurance). A recent integrative literature review showed that the major part of studies identified positive associations between self-reported health status in relation to patients’ satisfaction [69], particularly regarding nursing care. Previous evidence also supports the connection between healthcare effectiveness and patients’ perception of healthcare quality [13], particularly in the context of patient-centered health systems.

Findings of the present study showed that higher educational attainment and utilization of healthcare in public facilities presented a negative association with quality. Previous studies in Brazil showed a lack of consensus regarding the role of educational attainment in the perception of PHC quality in the context of the Family Health Strategy program. One ethnographic study conducted in five municipalities in the state of Bahia (northeast region) identified a positive association between educational attainment and patients’ views on quality of care [18], whilst another quantitative study in the municipality of Recife (state of Pernambuco, northeast region) showed a negative association between educational attainment and the evaluation of healthcare quality [21]. Nevertheless, the studies encompass evidence representative of a local level [18,21], whilst findings of the present investigation refer to population-based surveys representative of the national level.

Furthermore, the present study indicated a lack of significant association between certain individual and household-level characteristics (sex, skin color/ethnicity, occupational status, and household residents) and the perception of PHC quality. Similarly, evidence from an integrative literature review showed conflicting evidence or lack of significance in associations between the assessment of healthcare quality in relation to personal characteristics like educational attainment, income, and skin color/ethnicity [69]. Therefore, the empirical evidence shown in the present study emphasizes that key elements in promoting users’ satisfaction in the Brazilian health system are predominantly linked to health services and policy-level characteristics, i.e., the ability to solve patients’ health issues independently of severity, payment for performance, and source of healthcare funding. Thus, the findings highlight the need to focus on monitoring conditions of healthcare provision at a local level, including the supply and maintenance of adequate infrastructure and proper training and refresher courses for human resources training in PHC, particularly in public facilities, ensuring the promotion of improvements in patients’ health status.

## 5. Limitations and Contributions

This study presents certain limitations, particularly regarding the study design and data collection. First, the utilization of cross-sectional data hinders the establishment of causal relations in statistical analysis. Yet, the adoption of individual-level datasets with complex sampling selection procedures, ensuring population representativeness, allowed the identification of trends and factors associated with patients’ perception of PHC quality.

Second, the data collection based on self-reported information regarding health status and healthcare utilization potentially includes errors. In particular, overreporting of healthcare utilization may occur among individuals declaring poor health status and high satisfaction with health services [70]. Nevertheless, self-reported information on health has been an extensively validated tool in social sciences [71]. Furthermore, the adoption of empirical strategies including control variables for state of residence, year of the survey, and their interactions allowed us to obtain robust estimates, thus minimizing potential bias.

Third, the datasets included in the analyses comprise two types of population-level surveys, with the first type of survey being based on household sample with supplementary information on health status and healthcare utilization, and the second type of survey based on a national health survey focusing on health status and healthcare utilization. Yet, questionnaires adopted in both types of survey were based on identical questions from validated data collection tools and were conducted by the same government institution responsible for major surveys in Brazil, the Brazilian Institute for Geography and Statistics (Instituto Brasileiro de Geografia e Estatística, IBGE).

Fourth, healthcare quality was based on the patients’ views regarding quality of care received two weeks before data collection, thus comprising a subjective indicator that may be influenced by diverse contextual and personal characteristics. In addition, healthcare effectiveness was estimated through the appointments required to solve the individual’s health issue within the two-week period. Therefore, the indicators may be affected by the type of disease or health condition of the patient. However, the adoption of robust empirical strategy including variables to control for self-perception of health status and health issue severity driving PHC demand allowed for the identification of potential interferences of health characteristics in the analysis, thus minimizing potential bias in the findings.

Finally, it is important to highlight the contributions of the study to the field of knowledge, considering the scarcity of evidence on patients’ perception of primary healthcare quality in developing countries, particularly in Latin American countries [29]. Previous studies focusing on assessments of performance in primary healthcare within the Brazilian health system comprise specific evidence on primary healthcare quality representative at the local level [18,19,20,21,22,23,24].

## 6. Conclusions

The findings of the present study contribute to advances in the field of knowledge regarding patients’ satisfaction with PHC, showing its connections with individual, health system, and health service characteristics. Individual-level characteristics influencing the perception of PHC quality were predominantly linked to patients’ health conditions, whereas effects linked to health service characteristics indicated gaps in the public sector management of PHC activities. Evidence on health system characteristics showed that the implementation of payment for performance through the PMAQ-AB presented substantial positive effects on patients’ perception of healthcare quality. Thus, the study indicates that primary healthcare policies require additional incentives to foster the pursuit of quality in public facilities.

Yet, the lack of evidence-based decision-making in public policies of health in Brazil lead to the discontinuation of the PMAQ-AB following the third cycle of evaluation in 2019 and failures in the implementation of its successor, Previne Brasil, which may compromise further progress in achieving health equity within the country. Therefore, the findings of the study support the reinstatement of the PMAQ-AB or improvements in the implementation of payment for performance within the Previne Brasil program. Furthermore, actionable insights on the positive effects of financial incentives linked to performance in the assessment of primary healthcare quality within the SUS emphasize the need for institutional change in the Brazilian health system towards evidence-based decision-making in public policies of health.

## Figures and Tables

**Table 1:** Characteristics of variables in the dataset. Brazil, 1998–2019.

Variable	N	Frequency		Min	Max
		n	%		
Sex	136,441	90,893	66.62	0	1
Age ^‡^	136,429	45.67	17.41	18	108
Skin color/ethnicity					
Black	136,431	11,235	8.23	0	1
Brown	136,431	56,065	41.09	0	1
Indigenous	136,431	624	0.46	0	1
White	136,431	67,710	49.63	0	1
Yellow	136,431	797	0.58	0	1
Educational attainment ^‡^	129,609	8.69	5.14	0	18
Occupational status	134,189	71,578	53.34	0	1
Household residents ^‡^	167,624	3.12	1.92	1	25
Household income per capita in PPP ^‡^	164,935	805.03	1573.73	0.00	129,358.70
Health insurance ownership	136,402	50,557	37.06	0	1
Good health status	167,624	113,939	67.97	0	1
Multimorbidity	167,624	96,668	57.67	0	1
Type of healthcare facility					
Public	127,536	66,731	52.32	0	1
Private	127,536	60,522	47.45	0	1
Healthcare financing					
Health insurance	127,536	38,517	30.20	0	1
Out of pocket	127,536	26,315	20.63	0	1
SUS	127,536	62,704	49.52	0	1
Perception of good healthcare quality	167,624	164,193	97.95	0	1
Healthcare effectiveness ^‡^	131,372	96.93	7.74	7.14	100.00
Health issue severity	128,212	1199	0.94	0	1
Adherence to PMAQ-AB	167,624	66,381	39.60	0	1
Area	167,624	23,699	14.14	0	1

Obs.: N = valid observations in the dataset; n = absolute frequency; % = relative frequency; Min = minimum value; Max = maximum value. ^‡^ mean and standard deviation for continuous variables.

**Table 2:** Sociodemographic and economic characteristics of individuals, according to year of the survey. Brazil, 1998–2019.

Characteristics	1998	2003	2008	2013	2019	Total
Sex						
Male *	32.41	32.44	35.34	33.23	34.61	33.82
Female *	67.59	67.56	64.66	66.77	65.39	66.18
Age ^‡^	43.63	44.65	45.70	47.98	49.55	46.90
	0.34	0.13	0.12	0.24	0.19	0.10
Skin color/ethnicity						
Black *	5.81	6.26	7.46	8.57	10.94	8.32
Brown *	31.33	34.14	38.02	37.47	39.41	36.84
Indigenous *	0.27	0.20	0.36	0.49	0.48	0.39
White *	61.97	58.98	53.53	52.47	48.21	53.68
Yellow *	0.62	0.42	0.64	1.00	0.95	0.77
Educational attainment ^‡^	7.42	7.85	8.54	9.26	10.06	8.89
	0.24	0.06	0.05	0.09	0.07	0.03
Occupational status						
Employed *	53.13	52.72	56.79	51.67	50.79	52.81
Unemployed *	46.87	47.28	43.21	48.33	49.21	47.19
Household residents ^‡^	3.99	3.75	3.55	3.29	3.06	3.44
	0.05	0.02	0.01	0.02	0.02	0.01
Household income per capita ^‡^	933.89	795.36	925.92	1174.43	1277.08	1065.46
	84.14	13.44	13.61	41.98	26.53	15.32
Area						
Urban *	86.66	88.73	88.26	88.76	88.84	88.42
Rural *	13.34	11.27	11.74	11.24	11.16	11.58
Region						
North *	4.00	4.30	5.24	5.00	5.84	5.05
Northeast *	21.13	22.45	22.48	21.72	23.20	22.35
Southeast *	49.58	48.26	47.98	48.17	47.84	48.23
South *	18.81	18.52	17.61	18.61	16.37	17.77
Middle West *	6.48	6.47	6.69	6.52	6.75	6.61

Obs.: ^‡^ mean and standard error; * frequency.

**Table 3:** Health and healthcare utilization characteristics of individuals, according to year of the survey. Brazil, 1998–2019.

Characteristics	1998	2003	2008	2013	2019	Total
Good health status *	52.18	53.43	52.37	75.95	75.22	64.40
Multimorbidity *	38.77	36.36	36.49	70.32	70.04	54.02
Health insurance ownership *	41.25	38.67	37.42	37.35	38.65	38.44
Type of healthcare facility						
Public *	46.47	52.01	51.70	56.52	49.84	51.70
Private *	53.19	47.80	48.15	43.28	49.98	48.10
Unknown *	0.34	0.19	0.15	0.20	0.18	0.20
Healthcare funding						
Health insurance *	32.04	30.75	28.98	31.05	33.48	31.33
Out of pocket *	20.39	18.89	22.98	17.70	22.47	20.60
SUS *	40.35	48.99	49.77	55.56	48.93	49.52
Perception of good healthcare quality *	97.93	97.89	97.27	98.59	96.88	97.63
Healthcare effectiveness ^‡^	96.15	96.20	96.37	99.50	97.94	97.43
	0.08	0.06	0.06	0.05	0.07	0.04
Health issue severity *	0.67	0.80	0.86	0.47	1.83	0.99

Obs.: ^‡^ mean and standard error; * frequency.

**Table 4:** Pairwise comparisons and marginal predictions for perception of primary healthcare quality according to source of funding and type of healthcare facility. Brazil, 1998–2019.

Marginal Predictions—PHC Quality		1998	2003	2008	2013	2019
Public sector funding	Margin	0.965	0.963	0.954	0.981	0.948
	SE	0.003	0.002	0.002	0.002	0.003
Private sector funding	Margin	0.988	0.993	0.989	0.993	0.995
	SE	0.001	0.001	0.001	0.001	0.001
Pairwise comparison	Contrast	−0.023 *	−0.030 *	−0.035 *	−0.012 *	−0.047 *
	SE	0.003	0.002	0.002	0.002	0.003
Public facility	Margin	0.964	0.963	0.954	0.981	0.948
	SE	0.002	0.002	0.002	0.002	0.003
Private facility	Margin	0.992	0.994	0.990	0.992	0.995
	SE	0.001	0.001	0.001	0.001	0.001
Pairwise comparison	Contrast	−0.028 *	−0.031 *	−0.036 *	−0.011 *	−0.047 *
	SE	0.003	0.002	0.002	0.002	0.003

Obs.: SE = standard error; * *p* < 0.001.

**Table 5:** Odds ratios of logistic regression model. Brazil, 1998–2019.

Perception of Good Healthcare Quality		OR	SE	Sig.	95% CI
Healthcare effectiveness	(%)	1.017	0.002	***	1.013; 1.021
Adherence to PMAQ-AB	(yes = 1)	3.376	1.249	**	1.635; 6.971
Sex	(fem = 1)	0.901	0.058		0.794; 1.022
Age	(years)	1.008	0.002	***	1.004; 1.012
Educational attainment	(years)	0.981	0.008	*	0.965; 0.997
Good health status	(yes = 1)	3.209	0.216	***	2.813; 3.661
Multimorbidity	(yes = 1)	1.497	0.094	***	1.323; 1.694
Health issue severity	(yes = 1)	2.258	0.742	*	1.185; 4.301
Public healthcare facility	(yes = 1)	0.358	0.048	***	0.275; 0.467
Healthcare financed through health insurance	(yes = 1)	2.344	0.335	***	1.772; 3.102
Healthcare financed out of pocket	(yes = 1)	1.337	0.186	*	1.018; 1.757
Household income per capita	(yes = 1)	1.000	0.000	*	1.000; 1.000
Area	(rural = 1)	1.549	0.130	***	1.314; 1.826
Area under ROC curve		0.7694			
(95% CI)		(0.7614; 0.7774)			
Hosmer–Lemeshow χ^2^		1564.48			
Prob > χ^2^		0.1133			

Obs.: OR = odds ratio; SE = standard error; 95% CI = 95% confidence interval; *** *p* < 0.001; ** *p* < 0.01; * *p* < 0.05. Model includes control variables for state of residence, year of the survey, and interaction between state of residence and year of the survey.

## Data Availability

Datasets in the present study are publicly available on the platform of the Brazilian Institute for Geography and Statistics (Instituto Brasileiro de Geografia e Estatística, IBGE): PNAD (https://www.ibge.gov.br/estatisticas/sociais/populacao/19897-sintese-de-indicadores-pnad2.html?=&t=microdados accessed on 5 April 2025) and PNS (https://www.ibge.gov.br/estatisticas/downloads-estatisticas.html accessed on 5 April 2025).

## Appendix A

**Table A1:** Pairwise comparisons and marginal predictions for perception of primary healthcare quality according to remaining independent variables. Brazil, 1998–2019.

Marginal Predictions—PHC Quality		1998	2003	2008	2013	2019
Sex—Female	Margin	0.978	0.978	0.973	0.985	0.964
	SE	0.002	0.001	0.001	0.002	0.002
Sex—Male	Margin	0.981	0.981	0.973	0.988	0.978
	SE	0.002	0.002	0.002	0.002	0.002
Pairwise comparison	Contrast	−0.003	−0.003	0.000	−0.003	−0.014 *
	SE	0.002	0.002	0.002	0.003	0.003
Good health status	Margin	0.984	0.985	0.982	0.994	0.985
	SE	0.001	0.001	0.001	0.001	0.001
Less than good health status	Margin	0.974	0.972	0.962	0.960	0.920
	SE	0.002	0.002	0.002	0.004	0.005
Pairwise comparison	Contrast	0.108 *	0.012 *	0.020 *	0.035 *	0.065 *
	SE	0.002	0.002	0.002	0.004	0.005
Multimorbidity diagnosis—Yes	Margin	0.976	0.975	0.965	0.990	0.977
	SE	0.002	0.002	0.002	0.001	0.002
Multimorbidity diagnosis—No	Margin	0.982	0.981	0.977	0.976	0.949
	SE	0.002	0.001	0.001	0.003	0.004
Pairwise comparison	Contrast	−0.006 ^‡^	−0.006 ^‡^	−0.012 *	0.015 *	0.028 *
	SE	0.002	0.002	0.002	0.003	0.004
Health issue severity—High	Margin	0.966	0.990	0.992	0.998	0.990
	SE	0.015	0.006	0.005	0.002	0.005
Health issue severity—Low	Margin	0.979	0.978	0.972	0.986	0.972
	SE	0.001	0.001	0.001	0.001	0.002
Pairwise comparison	Contrast	−0.013	0.012	0.021 *	0.012 *	0.018 ^‡^
	SE	0.015	0.006	0.005	0.002	0.006
Rural area	Margin	0.981	0.986	0.979	0.986	0.968
	SE	0.003	0.002	0.003	0.332	0.004
Urban area	Margin	0.979	0.978	0.972	0.986	0.969
	SE	0.002	0.106	0.001	0.001	0.002
Pairwise comparison	Contrast	0.002	0.008 ^‡^	0.007 ^§^	0.000	−0.001
	SE	0.004	0.002	0.003	0.004	0.004

Obs.: SE = standard error; * *p* < 0.001; ^‡^ *p* < 0.01; ^§^ *p* < 0.05.
